# Conditional anterograde tracing reveals distinct targeting of individual serotonin cell groups (B5–B9) to the forebrain and brainstem

**DOI:** 10.1007/s00429-014-0924-4

**Published:** 2014-11-18

**Authors:** Aude Muzerelle, Sophie Scotto-Lomassese, Jean François Bernard, Mariano Soiza-Reilly, Patricia Gaspar

**Affiliations:** 1 Inserm UMR-S 839, 17 rue du Fer à Moulin, 75005 Paris, France; 2 Université Pierre et Marie Curie, Paris, France; 3 Institut du Fer à Moulin, Paris, France; 4 Inserm UMR- 894, Centre Psychiatrie St Anne, Paris, France

**Keywords:** Genetic mouse models, Anatomical tract-tracing, GFP, SERT, Serotonin, Tegmental nucleus, Pearson correlation, Olfactory bulb, Habenula, Prefrontal cortex, Median raphe, Dorsal raphe, Hippocampus, Amygdala, Axon tracing, AAV

## Abstract

Serotoninergic innervation of the central nervous system is provided by hindbrain raphe nuclei (B1–B9). The extent to which each raphe subdivision has distinct topographic organization of their projections is still unclear. We provide a comprehensive description of the main targets of the rostral serotonin (5-HT) raphe subgroups (B5–B9) in the mouse brain. Adeno-associated viruses that conditionally express GFP under the control of the 5-HT transporter promoter were used to label small groups of 5-HT neurons in the dorsal (B7d), ventral (B7v), lateral (B7l), and caudal (B6) subcomponents of the dorsal raphe (DR) nucleus as well as in the rostral and caudal parts of the median raphe (MR) nucleus (B8 and B5, respectively), and in the supralemniscal (B9) cell group. We illustrate the distinctive and largely non-overlapping projection areas of these cell groups: for instance, DR (B7) projects to basal parts of the forebrain, such as the amygdala, whereas MR (B8) is the main 5-HT source to the hippocampus, septum, and mesopontine tegmental nuclei. Distinct subsets of B7 have preferential brain targets: B7v is the main source of 5-HT for the cortex and amygdala while B7d innervates the hypothalamus. We reveal for the first time the target areas of the B9 cell group, demonstrating projections to the caudate, prefrontal cortex, substantia nigra, locus coeruleus and to the raphe cell groups. The broad topographic organization of the different raphe subnuclei is likely to underlie the different functional roles in which 5-HT has been implicated in the brain. The present mapping study could serve as the basis for genetically driven specific targeting of the different subcomponents of the mouse raphe system.

**Electronic supplementary material:**

The online version of this article (doi:10.1007/s00429-014-0924-4) contains supplementary material, which is available to authorized users.

## Introduction

Serotonin (5-hydroxytryptamine, 5-HT) is a neuromodulator with a wide variety of identified roles in brain functions as diverse as learning, perception, neuro-vegetative, neuro-endocrine, or mood control (Lucki 1998). These widespread effects have raised the question of the structural counterpart of these diverse functions (Calizo et al. 2011; Hale et al. 2011; Kiyasova et al. 2011; Gaspar and Lillesaar 2012). Not surprisingly, given the numerous neuronal functions of 5-HT, there is hardly no region in the central nervous system that does not receive 5-HT innervation (Jacobs and Azmitia 1992; Steinbusch 1981). In mammals, the extensive 5-HT axonal network is entirely derived from small groups of neurons located in the hindbrain. The total number of the 5-HT neurons is small, 28,000 neurons in mice (Ishimura et al. 1988), in comparison with the vast brain territory that they innervate, suggesting a highly complex and divergent organization. Moreover, individual 5-HT neurons are highly collateralized (Fallon and Loughlin 1982; Gagnon and Parent 2014), and their axons have a large number of varicosities allowing non-synaptic release (Beaudet and Descarries 1981). These anatomical and physiological features led to the concept of volume transmission, promoting the view that the topographic organization of the 5-HT systems is not crucial for their modulatory role (Fuxe et al. 2010). Yet a substantial amount of evidence points to some form of topographic organization of the 5-HT systems across many species. In invertebrates such as lobsters, 5-HT neurons have a metameric organization, with each 5-HT neuron projecting to different sensory ganglia (Ma et al. 1992); in C. Elegans, each of the five 5-HT neurons has a distinct projection domain (Zheng et al. 2005). In fish, several groups of 5-HT neurons in the diencephalon and mesencephalon also have distinct projections (Lillesaar 2011; Lillesaar et al. 2009; Gaspar and Lillesaar 2012). In the mammalian brain, many studies using different anatomical tracing methods have identified a topographic organization of the raphe neurons. Specifically, studies in rats allowed to distinguish differential projections from the caudal (B1–B3), the median/central raphe (MR) groups (B5, B8), and the dorsal raphe (DR) cell groups (B6, B7) (Azmitia and Segal 1978; Bobillier et al. 1976, 1979; Jacobs et al. 1974, 1978; Vertes 1991; Vertes et al. 1999). Moreover, a local rostrocaudal organization within the DR was described (Imai et al. 1986). However, a limitation of most of previous anatomical analyses concerns the tracers utilized (e.g., tritiated amino acids or phaseolus) that did not allow the unambiguous identification of the 5-HT projections. Indeed 5-HT neurons are generally intermingled with larger populations of non 5-HT neurons in each raphe subnuclei. An example of this is the DR cell group, which contains the highest proportion of 5-HT producing neurons, and yet comprises twice as much non-5-HT neurons (Descarries et al. 1982). Typically, the identification of 5-HT projections from the raphe relied on tracing techniques coupled to the histochemical revelation of 5-HT or combined with lesions. Although these studies allowed to describe the projections of the raphe cell groups, they generally focused on a particular target brain region or at most on a combination of few targets (Jones and Cuello 1989; Kohler et al. 1982; Kohler and Steinbusch 1982; Van Bockstaele et al. 1993; van der Kooy and Hattori 1980; Vasudeva et al. 2011; Waterhouse et al. 1986). It is, therefore, difficult at present to have a comprehensive picture of the different targets of a given subgroup of raphe cells. More recent genetic studies defined the rhombomeric origin of the different raphe nuclei (Jensen et al. 2008) and provided a useful description of the 5-HT axon projections arising from the different rhombomeres (Bang et al. 2011). However, this approach does not allow a specific delimitation of the topographic organization of raphe neurons originating from the same rhombomere (Jensen et al. 2008).

The advent of new genetic techniques in mice, in particular of viral vectors allowing the delivery of optogenetic tools or designer receptors exclusively activated by designer drugs (DREADDs), makes it necessary to have more accurate maps of the projection fields of a given raphe 5-HT cell subgroup to dissect out more precisely the selective functional role of subsets of 5-HT neurons in different brain functions. For this, we used conditional tracing methods (AAV, lox-stop-lox-GFP) in mice expressing the Cre-recombinase in 5-HT raphe neurons to obtain a selective anterograde tracing of these neurons. Using this method, we labeled small groups of 5-HT neurons within the different raphe subnuclei allowing a comprehensive mapping of their terminal axon fields in the brain. In the following account we provide a description of the main projecting areas of 5-HT axons arising from different raphe subnuclei as well as a systematic semi-quantitative analysis of correlations between the origin of 5-HT axons (raphe subnuclei) and their targeted brain regions.

## Materials and methods

### Animals

All experiments were performed in mice in compliance with the standard ethical guidelines (European Community Guidelines and French Agriculture and Forestry Ministry Guidelines for Handling Animals decree 87849).

The mouse lines, maintained on a C57-Bl6 background are locally bred and maintained under standard laboratory conditions (22 ± 1 °C, 60 % relative humidity, 12–12 h. light–dark cycle, food and water ad libitum). After weaning, male and female were housed separately (4–5 animals per cage).

The serotonin transporter (SERT, Slc6a4) Cre mouse line was used in most experiments. This is a knock-in of the nls-Cre in the 5’UTR region of the SERT gene (Slc6a4tm1(cre)Xz) as previously described (Narboux-Neme et al. 2008; Zhuang et al. 2005). The original strain has been backcrossed on a C57-bl6-J background for more than 5 generations, and will be referred to as SERT^cre^ in the following account. In this mouse line, Cre-recombinase expression follows precisely the temporal expression of the SERT gene: expression is broader than the raphe before P14, however, in adults SERT-Cre driven expression is only present in the 5-HT neurons of the raphe and shows a highly efficient recombination. All tracing experiments were done on SERT^Cre/+^ mice obtained by crossing SERT^cre/cre^ male mice with C57-bl6-J mice.

**Fig. 1:**
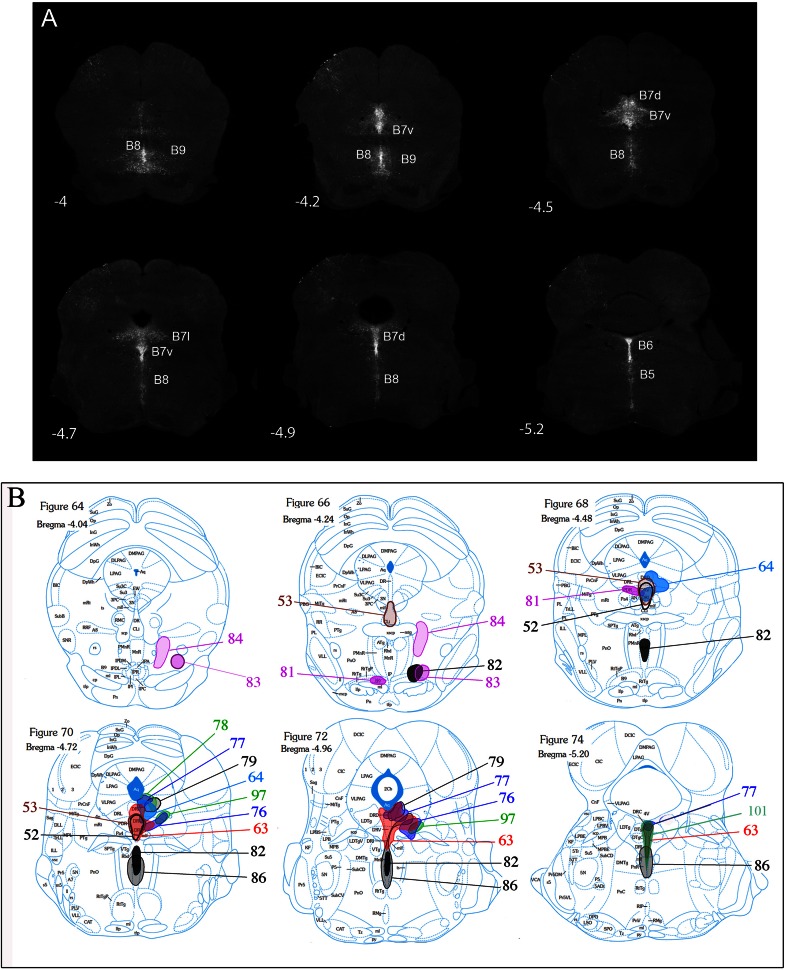
Nomenclature of the raphe subnuclei and main stereotaxic injection sites. **a** Shows the localization of the main serotonin cell groups that were analyzed in the present study. For this illustration we used GFP-immunostained serial sections through the brainstem obtained from a P28 Pet1-Cre::GFP mouse (B6.Cg-Tg(Fev-cre)1Esd/J). Figure **b** is a schematic rendering of the localization of GFP-transfected neurons in the different cases described in the following account. They have been matched to the corresponding planes of the Paxinos mouse atlas (Paxinos and Franklin 2001). For clarity, not all cases are illustrated but the main raphe subnuclei targeted in the cases for Pearson correlation are available in supplementary Table 1

Because SERT^Cre/+^ cannot be used for developmental analyses of the raphe, due to extra raphe expression, we used the Pet1^Cre^ (Scott et al. 2005; Jackson lab name strain: B6.Cg-Tg(Fev-cre)1Esd/J) crossed to the RCE-lox-stop-GFP mouse line that conditionally expresses GFP under the control of the Rosa promoter. This enables a developmental recombination that permanently and exclusively labels all 5-HT raphe neurons. This mouse line was only used to illustrate the localization of the 5-HT cell groups in the rostral raphe (Fig. 1a).

C57-Bl6-J mice (CER-Janvier, France) were used to control for Cre recombination specificity after viral injections.

All tracing experiments were carried out on P60–P120 mice (20–30 g) of both genders (male and female in equal numbers).

### Viral tools

The replication-defective adeno-associated viruses (AAV) from Penn Core Vector (University of Pennsylvania, USA) were used to obtain a conditional GFP expression in the 5-HT raphe neurons. In the majority of our experiments, we used the AAV2/1.CAG.LSL.EGFP.bGH (Allen Institute 851, lot 1676) and the AAV2/9.CAG.LSL.EGFP.bGH (Allen Institute 851, lot 1681) in which the genome is based on recombinant AAV2, while the capsid is based on AAV1 or AAV9. Both allow the GFP expression under the CAG promoter after Cre excision of the LoxP-Stop-LoxP (LSL) sequence.

A few pilot experiments utilized the AAV1.CAG.FLEX.EGFP.WPRE.bGH (catalog reference AV-1-ALL854) from Penn Core Vector (University of Pennsylvania, USA) in which a flip-excision (FLEX) switch allows expression of the GFP only if the LSL sequence is excised and the inverted coding sequence of the GFP is aligned with the promoter sequence.

The titration of these AAV is within 10^12^–10^13^ genome containing particles/ml.

### Stereotaxic injections targeting raphe nuclei subgroups

Experimental mice were anesthetized with ketamine (150 mg/kg)/xylazine (10 mg/kg) (Sigma-Aldrich Co., MO, USA) before surgery. To maintain the skull flat, the palate bar was adjusted after measuring the dorsoventral bregma and lambda positions. The medio-lateral arm of the stereotaxic apparatus (David Kopf Instrument, CA, USA) was generally maintained vertical, except when targeting the B7 raphe nucleus which required a slope of 10° angle (Table 1). Single injections (10–25 nl) were performed using a pulled glass capillary (30 to 50 µm tip diameter; PCR micropipette, Drummond Scientific company) fixed to an adapter specially designed to be mounted on the oil hydraulic micromanipulator MO-10 (Narishige, Japan). Red beads (Lumafluor Inc, USA) were added to the AAV solution (1/7 vv) to visualize the full trace of the injection site. The capillary was left in the target site for 5 min after injection to prevent backflow. The coordinates used to target the different raphe nuclei subgroups are summarized in Table 1.

**Table 1:** Stereotaxic coordinates used to target the different raphe subnuclei

Raphe nucleus labeled	Case numbers	Stereotaxic coordinates		
		AP from bregma or lambda	ML	DV
B7d + v + l	S63^a^, S64^a^	0.5 from lambda	1	−2.9
B7v	S52^a^, S53^a^	0.5 from lambda	1	−3.2
B7d + l	S3^a^, S4^a^, S77, S78, S79	0.5 from lambda −4.9 from bregma	0.8 0.75	−3.2 −2.8
B7 l	S97	−4.9 from bregma	0.9	−2.8
B6–B5	S101, S146, S161	−5.2 from bregma	0.5	−2.4
B9–B8	S82, S86, S163	−3.9 from bregma	0.6 or 1.3	−4.2
B9	S83, S84, S110, S111, S153	−3.5 to −3.7 to bregma	0.6 or 1	−4.2

The main focus of the injection site is indicated for each case

*AP* antero-posterior, *ML* medio-lateral, *DV* dorsoventral (as measured from the cortical surface)

^a^Slope of 10° angle

### Tissue processing

Histology procedures were started 3 weeks after surgery. Anesthetized (Pentobarbital 0.5 mg/g) mice were fixed by intracardiac perfusion of 4 % paraformaldehyde in 0.1 M phosphate-buffered saline (PBS; pH 7.4). Brains were post-fixed overnight in the same fixative solution and cryoprotected during 2 days in 30 % sucrose containing sodium azide (0.01 %; Sigma-Aldrich Co., MO, USA). Coronal sections were prepared (50 µm thick) on a cryo-microtome (Microm Microtech, France). Serial sections from the olfactory bulb to the medulla were collected as series of 6, except for sections containing the raphe nuclei that were collected as series of 3. Tissues were stored at −20 °C in cryoprotectant (30 % ethylene glycol and 30 % glycerol in 0.12 M phosphate buffer) if not used immediately.

Free-floating sections were washed in three PBS baths before immunohistochemical procedures. To reduce non-specific binding, all the washes and antibody incubations were performed in blocking solution containing 2 g/L gelatin (Merck) and 0.25 % triton in PBS (PBGT). Primary antibodies were applied for 48 h at 4 °C, and secondary antibodies were applied for 2 h at room temperature (Table 2).

**Table 2:** List of antibodies used in this study, providers references and dilutions used in are indicated

Antibody	Provider	Reference	Dilution
Rabbit anti-GFP	Molecular Probes	A6455	1/2,500
Chicken anti-GFP	Aves Labs, Inc, USA	GFP-1020	1/1,000
Goat anti-SERT	Santa Cruz Biotechnology	Sc-1458	1/5,000
Rabbit anti-5-HT	Calbiochem	PC177L	1/5,000
Chicken anti-TH	Aves Labs, Inc, USA	TYH	1/1,000
Biotinylated Goat anti-rabbit	Vector Laboratories	BA-1000	1/200
488 Donkey anti-chicken	JacksonImmunoResearch, Europe Ltd	703-545-155	1/200
Cy3 Donkey anti-goat		705-165-147	1/200
Cy5 Donkey anti-rabbit		711-605-152	1/200

All these antibodies have previously been tested for specificity

DAB-peroxidase immunostaining of GFP was systematically performed on one complete series of sections. Endogenous peroxidase activity was quenched by incubating sections for 1 h in 1 % H_2_O_2_ diluted in PBGT prior to exposition to a rabbit anti-GFP antibody. After that, sections were incubated with a biotinylated-goat anti-rabbit, then washed in PBS and exposed to the streptavidin–horseradish peroxidase complex for 90 min (1:400 in PBS; GE Healthcare Life Science, NJ, USA). Revelation was adapted from (Bonnavion et al. 2010). Sections were first rinsed for 10 min in Tris–HCl (0.05 M, pH 7.6), and then immersed in a Tris–HCl solution containing 0.02 % diaminobenzidine (DAB, Sigma-Aldrich Co., MO, USA) and nickel ammonium sulfate (0.6 % Sigma-Aldrich Co., MO, USA). Subsequently, this solution was supplemented with increasing concentrations of H_2_O_2_ (from 0.00015 to 0.0012 %; Sigma-Aldrich Co., MO, USA) until optimal labeling was obtained. This reaction was stopped by three 10 min rinses in Tris–HCl and sections were washed in PBS, mounted on glass slides, air dried (24 h), dehydrated in a graded series of alcohol, cleared in xylene, and coverslipped with Eukitt (Sigma-Aldrich Co., MO, USA).

To verify specificity of GFP expression in 5-HT cell bodies and axons, double immunofluorescent labeling was carried out using primary antibodies against GFP (in chicken) with either SERT or 5-HT (see Table 2). In addition, projections of 5-HT axons to catecholaminergic nuclei were verified in some cases with double immunofluorescent labeling using primary antibodies against GFP (in rabbit) and TH (see Table 2). The corresponding fluorescent secondary antibodies were whole IgG all raised in donkey. Sections were finally mounted in Mowiol (10 %, Calbiochem, Germany)-Dabco (2.5 %, Sigma-Aldrich Co., MO, USA).

### Image acquisition and analysis

DAB-revealed sections were imaged using a slide scanner (Nanozoomer 2.0-HT C9600, Hamamatsu, Japan) objective X20 and analyzed with the NDP View2 software (Hamamatsu, Japan). This enabled fast mapping of GFP-axonal projection throughout the brain as it offers panoramic screening of whole tissue sections with fully electronic manipulation for the visualization and analysis of any individual regions (Zheng et al. 2014).These scanned slides are available online (Supplementary figure)

For illustration purposes, bright-field images were either exported in Tiff format from the nanozoomer images with NDP viewer, or captured with a Cool SNAP camera mounted on a provis microscope (Olympus, France).

For co-localization analyses, images were acquired on a Leica SP5 confocal system, equipped with an Argon laser (for the 488 nm excitation), a diode 561 nm and HeNe 633 nm. Z serie stacks of confocal images were acquired at 1024 × 1024 pixel resolution, with a pinhole set to one Airy unit and optimal settings for gain and offset. Identities of GFP-expressing cell body and axons were analyzed with a 40X/1.25 N.A plan-apochromat and a 63X/1.4 N.A plan-apochromat, respectively.

All cases were analyzed for localization and extent of injection. Some cases were discarded from further analyses either when there was an incomplete GFP immunolabeling of neurons with weak labeling of axon terminals, or when there was a too extensive labeling (e.g., labeling of both the DR and MR neurons).

Sections from the rostralmost to the caudalmost levels were examined independently by 3 investigators with the aid of both bright-field microscope and NDP images for GFP + fiber localization and density. A qualitative rating was made as follows: (+++) strong density of fibers, (++) moderate density of fibers, (+) few fibers (<10), (±) rare fibers (±2–3) and (-) no visible fibers. Labeled terminals were distinguished from fibers of passage by the presence of varicosities and the degree of axonal tortuosity. Subsequently, subjective rating was converted into a numerical scale (from 4 to 0, respectively) to further analyze correlations between the origin of 5-HT axon projections involving different groups of 5-HT neurons (raphe subnuclei) and their targeted brain regions. This was done by calculating the Pearson’s correlation coefficient using IBM SPSS Statistics 20 (IBM, NY, USA). Significance levels were established at *p* < 0.05.

Schematic rendering of the main injection sites was produced using Adobe illustrator. Relevant levels were extracted for the Paxinos Atlas (Paxinos and Franklin 2001) and drawings made with reference to the macroscopic photographs of the serial forebrain/brainstem sections.

## Results

### Nomenclature of the raphe subnuclei and localization of the main injection sites (Fig. 1)

The localization of the main 5-HT raphe cell groups targeted by stereotaxic injections is shown in Fig. 1a with a schematic outline of the principal injection sites (Fig. 1b, cell groups targeted in all cases are shown in supplementary Figure 1, http://1drv.ms/1y2FNst). The nomenclature of the 5-HT cell groups relies on the initial numbering system defined in the seminal description of 5-HT neurons in Dahlstrom and Fuxe (1964) and further subdivisions described in subsequent reports (Steinbusch and Nieuwenhuys 1981). Correspondence with the anatomical subgroups relied on detailed anatomical reviews (Hale and Lowry 2011; Tork 1990) as well as on more recent fate maps describing the embryonic origin of raphe cell subgroups (Alonso et al. 2012; Jensen et al. 2008).

The dorsal raphe nucleus (DR) extends over 1.2 mm in the rostrocaudal dimension including B7 rostrally and B6 caudally (Fig. 1a). In rats, up to six subgroups have been identified based on topographic and morphological criteria (Steinbusch and Nieuwenhuys 1981). In mice, we identified four main clusters that were targeted for tracer injections: the ventral cluster (referred to as B7v or DRv in the following account) that has Y shape just above and between the medial longitudinal fasciculus (mlf); the dorso-median cluster (B7d; DRd), that lies above the DRv, below the cerebral aqueduct; the lateral wings (B7l; DRl), a loosely aggregated group of cells lying lateral to the B7d. Finally, there is a caudal cluster (B6) that is in the caudal continuation of the B7d and is particularly prominent at the level of the enlargement of the fourth ventricle (Fig. 1a, Bregma level −5.2). Recent fate mapping studies indicated that both B7 and B6 neurons derive from rhombomere 1 (Jensen et al. 2008) with the most rostral midbrain components having an isthmic origin (Alonso et al. 2012).

The median raphe nucleus (MR) includes the B8 and B5 cell groups. From rostral to caudal: the caudal linear nucleus (Cli) that has an isthmic origin (Alonso et al. 2012), the rostral and caudal MR that are derived, respectively, from R1, R2 and R3 according to lineage studies (Alonso et al. 2012; Jensen et al. 2008). In the present study, the rostral (B8) and caudal (B5) components of the MR were selectively targeted, however, we were unable to obtain exclusive B5 labeling (Fig. 1b).

The B9 cell group is a numerically large 5-HT cell group (Vertes and Crane 1997) that has a more diffuse organization than the DR or MR. Scattered neurons of B9 are located lateral to the midline in the supralemniscal and pontomesencephalic reticular formation (Fig. 1a, Bregma levels −4 to −4.2). The embryonic origin of the B9 group is diverse as it appears to be derived from the 3 most rostral rhombomeres (named R1 sul, R2 sul, R3 sul) (Alonso et al. 2012). In the present study, only the most rostral and dorsal component of B9 was targeted (Fig. 1b).

**Fig. 2:**
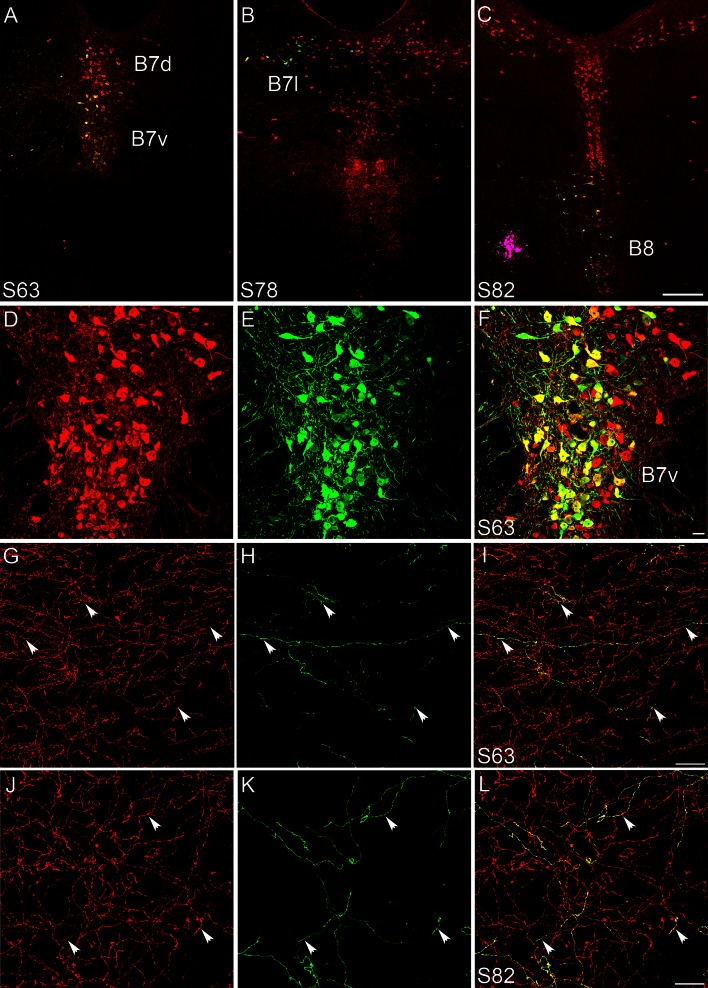
Selective labeling of sparse populations of 5-HT raphe neurons with conditional AAV viruses. Coronal sections through the raphe and target brain areas were co-immuno-labeled with anti-GFP (*green*) and 5-HT antisera (*red*). **a**–**c** The injection site sites of 3 representative cases is shown, in the B7v (**a**), the B7l (**b**), and the B8 (**c**). **d–f** shows a higher magnification of case S63 shown in **a**, with 5-HT (**d**), GFP (**e**), and merge of the two channels (**f**), showing that almost all GFP-expressing neurons are serotoninergic. **g**–**l** Double immunolabeling of GFP-5-HT labeled raphe axons after an injection in the DRv (**g**–**i**) and in the MR (**j**–**l**); showing that a large majority of the GFP-positive axons contain 5-HT, while only a small fraction of the total serotoninergic axon terminal network are labeled with GFP. *Scale bars* are 200 μm (**a**–**c**), 20 μm (**d**–**f**), and 20 μm (**g–l**)

In preliminary studies, we noted that the virus had a much larger diffusion radius than the fluorescent beads that were co-injected, resulting in a much larger site of infection than the desired targeting site. This spread is likely due to uptake of the viral particles by local dendrites that generally extend broadly, being within the diffusion sphere of the injection. Stereotaxic coordinates (indicated in Table 1) were, therefore, adjusted accordingly, to be slightly “off-target” by approximately 200 µm (see Fig. 2c) and the injection volume was reduced as much as possible. Although this had the inconvenience of resulting in a large amount of cases with insufficient labeling, this strategy was followed as the best trade-off for obtaining relatively small injection sites that were sufficiently restricted within one raphe subnucleus (Fig. 2a–c). In contrast we observed no neurons GFP-labeled outside the raphe nuclei, indicating a lack of recombination by passing axons or terminals that may have taken up the virus at the injection site.

Twenty cases (supplementary Table 1) with injection sites of comparable sizes and clear labeling of axon terminals were selected for systematic semi-quantitative correlation analysis between injections sites (raphe subnuclei) and forebrain and brainstem axon targets.

### Specificity of the conditional AAV labeling (Fig. 2)

To determine the selectivity of the labeling, we systematically carried out a combination of triple labeling using GFP, 5-HT and SERT on one series of sections. This showed that the large majority of the GFP-labeled neurons contained 5-HT (Fig. 2a–f). However, in some cases, GFP-labeled glial and neuronal cells that did not contain 5-HT were also observed, (Fig. 8c). Additionally, non-specific labeling was noted in Cre-negative control *C57-bl6-J* mice (data not shown) indicating some degree of “leakage” of the floxed GFP viral construct rather than spurious Cre-recombinase expression. A similar leakage was observed when using the AAV-Flex viruses that were used for comparison purposes. The non-5HT GFP expression was mainly found in glial cells (Fig. 8c) and short distance projecting neurons, but was also noted in a variable proportion non-5-HT axons ascending in the median forebrain bundle (mfb) (1 to 10 % according to the cases). We, therefore, systematically carried out additional control of double labeling in the terminal fields described to confirm the co-localization of GFP and SERT (Fig. 2g–l). The following description of target projections refers only to these validated instances.

In addition to fluorescent labeling, we systematically processed one series of sections with DAB nickel-enhanced revelation to increase the visibility of the fine caliber axon terminals in the distal projection areas. With this intensification procedure, even weak GFP signals are detected, decreasing the risk of false-negative results. Furthermore, such chromogenic revelation eased the systematic high-throughput acquisition of images with the Nanozoomer microscope and facilitated the comparative evaluation by different observers.

### DR projections (B7)

The description of the DR projections is based on the analysis of 9 selected cases, with either large injections involving several DR subcomponents (as illustrated for cases S63 and S64 in Figs. 1b, 2a), or cases with more restricted labeling circumscribed to the ventral, lateral (Fig. 2b), dorsal, or caudal subcomponents of the DR (Fig. 1b).

All labeled axons arising from DR cases had ascending projections in the mfb and a variable degree of descending projections to the pons and medulla. The terminal projections varied according to the DR subnuclei that were targeted as described in the following sections that describe the most typical cases for each location.

### Ventral component of DR (B7v) (Figs. 3, 4) (Table 3)

**Fig. 3:**
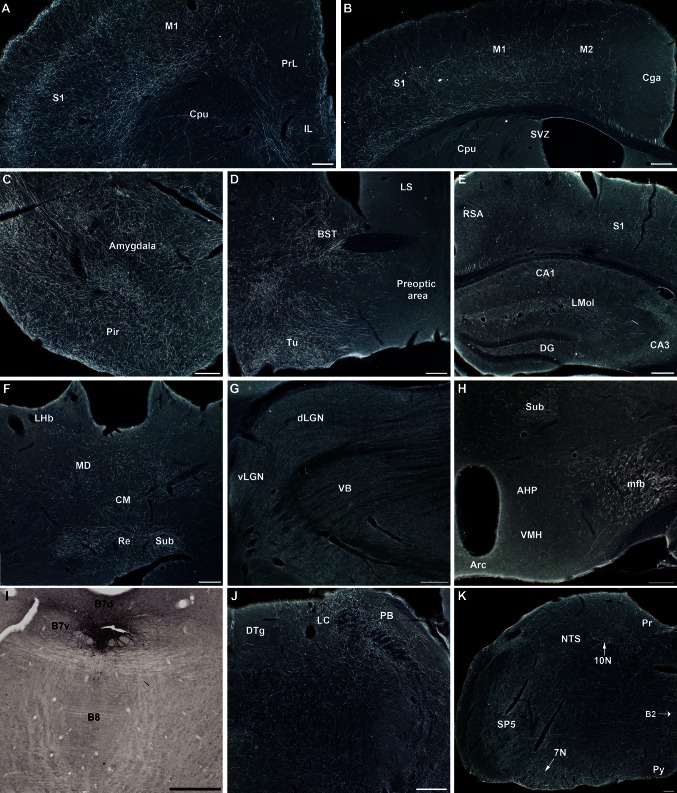
B7v 5-HT innervation to the forebrain and hindbrain (Case S53). GFP immunolabeling from coronal sections was revealed with enhanced DAB-nickel staining. Negative images of the micrographs were obtained by color conversion in Photoshop. **a**, **b** In the frontal cortex a high density of GFP-labeled terminal network is noted in the somatosensory (*S1*) and motor (*M1*) cortices compared to the medial prefrontal cortex (*Prl*) and anterior cingulate cortex (*Cga*). In the caudate (*Cpu*), limited patches of axon terminals are observed. **c** Amygdala and piriform (*Pir*) cortex are the most densely innervated structures from B7v serotonin neurons. **d** Preoptic area and septal areas are hardly innervated, whereas the BST and olfactory tubercle receive a very dense B7v innervation. **e** Hippocampus showed only scattered terminal innervation in the stratum lacunosum (*Lmol*). **f**–**g** In the thalamus, the innervation from the B7v is limited to midline cell groups, such as the nucleus reuniens (*Re*). In the habenula, a moderate innervation is visible in the lateral part (*LHb*). **h** Hypothalamic cell groups are hardly innervated by the B7v innervation despite the proximity of the labeled axons ascending in the medial forebrain bundle (*mfb*). **i** GFP-transfected neurons in the raphe are limited to the B7v with no neurons in the B7d or the MR. **j**, **k** In the brainstem B7v innervation is mainly found in the locus coeruleus (*LC*), the parabrachial nucleus (*PB*), and several nuclei of the cranial nerves (VII, X). *Scale bars* 100 μm. *AHP* anterior hypothalamic area, *CM* central medial thalamic nucleus, *DTg* dorsal tegmental nucleus of Gudden, *MD* mediodorsal thalamic nucleus, *mfb* medial forebrain bundle, *NTS* nucleus of the solitary tract, *Py* pyramidal tract, *RSA* retrosplenial area, *SP5* spinal trigeminal nucleus, *Tu* olfactory tubercle, *VB* ventrobasal thalamic nucleus

Two cases were chosen to illustrate the B7v projections (S52, S53), in which the GFP-labeled neurons were restricted to the most ventral part of B7 (Figs. 1b, 3i) and displayed almost identical bilateral projection patterns.

**Fig. 4:**
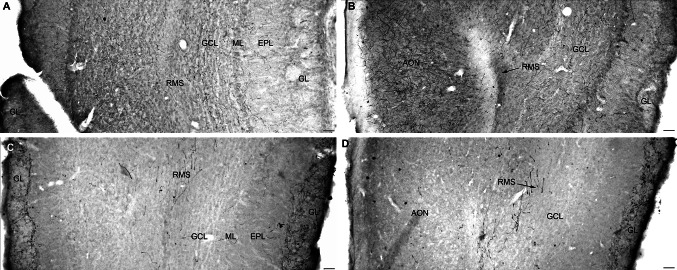
Complementary innervation from the B7v (**a**, **b**) and MR (**c**, **d**) in the olfactory bulb (OB) (Cases S53 and S156). GFP immunolabeling is shown at a rostral (**a**, **c**) and a caudal (**b**, **d**) level of the olfactory bulb. The granular (*GCL*) and the mitral (*ML*) layers received 5-HT innervation from the B7v, whereas the periglomerular layer (*GL*) is exclusively innervated by the MR neurons. Anterior olfactory nucleus (*AON*) received 5-HT innervation from B7v (**b**), but not from MR raphe neurons (**d**). Interestingly, some 5-HT fibers were also detected in the rostral migratory stream (RMS), at the two OB levels analyzed, and only in the case where B8 was targeted (**c**, **d**), but not when B7 was targeted (**a**, **b**). *Scale bars* 50 μm

**Table 3:** Distribution of 5-HT projections from different raphe subgroups

Case number	S52, S53	S97	S3, S77, S78, S79	S101, S146	S82, S86	S84, S110
	(B7v)	(B7 l)	(B7d)	(B6)	(B8)	(B9)
Forebrain						
Olfactory bulb	**+++**	**+**	**+**	−	**++**	−
n. Accumbens	**+**	−	**+**	−	**++**	−
n. Stria terminalis	**+++**	−	**±**	−	**+**	−
Caudate-putamen	**+**	−	**±**	**+**	**±**	**++**
Lateral septum	−	−	**±**	**++**	**+++**	**+**
Medial septum	**++**	−	**+**	**++**	**+**	**++**
Amygdala						
Cortical	**+++**	−	**±**	**+**	−	−
Basolateral	**+++**	−	**+**	−	**+**	−
Central	**++**	−	**+**	−	**±**	−
Medial	**+**	−	**++**	−	**±**	**+**
Cortex						
Orbital	**++**	−	−	−	**±**	−
Prefrontal	**++**	−	**±**	−	**+**	**±**
Entorhinal	**+++**	**+**	**+**	**+**	−	**±**
Piriform	**+++**	**+**	**+**	−	−	−
Insular	**+++**	−	**±**	−	−	**±**
Cingulate	**±**	−	−	**+**	**+**	−
Sensory	**+**	**±**	−	**++**	**±**	**±**
Motor	**+**	−	−	**+**	**+**	−
Temporal	−	−	−	−	**±**	−
Thalamus						
Lateral	**±**	**+++**	**++**	−	**±**	−
Medial	**+++**	**++**	**+**	−	**+**	−
Lateral habenula	**+**	**+**	**+**	**+**	**±**	−
Medial habenula	**+**	**+**	**+**	**+**	**++**	−
Paraventricular n.	**+**	**+**	**+**	−	**+++**	**+**
Hypothalamus						
Anterior	**+**	**+**	**++**	**+**	**++**	**+**
Preoptic	**±**	−	**++**	**++**	**+++**	**±**
Mammillary n.	**±**	**++**	**+**	−	**++**	**+**
Arcuate n.	−	−	**±**	−	**++**	−
Hippocampus						
CA	**±**	−	**–**	**++**	**+++**	−
Dentate gyrus	−	−	**–**	**++**	**+++**	−
Subiculum	−	−	**±**	**+**	**++**	−
Brainstem						
Parabrachial n.	**++**	**+**	**±**	**+**	**++**	**+**
Locus coeruleus	**++**	−	**+**	**±**	**+**	**++**
Periaqueductal gray	**+**	**+**	**+**	**+**	**±**	**++**
Ventral tegmental area	−	−	**+**	**+**	**+**	**++**
Substantia nigra	**++**	−	**+**	**±**	**+**	**+**
Superior colliculus	**+**	**+**	**+**	−	**++**	**±**
Cerebellum	−	**+**	**±**	−	**+**	−
Dorsal tegmental n. (Gudden)	−	−	**–**	−	**+++**	−
Interpeduncular n.	−	−	**–**	**±**	**+++**	**±**
Prepositus n.	**++**	**+++**	**+**	−	**++**	**±**
Trigeminal nerve	**++**	−	**±**	**+**	−	**±**
Olivocochlear nerve	**++**	−	**±**	**+**	−	**±**
Deep cerebellum	**+**	**++**	**–**	**+**	**±**	**++**
Raphe magnus (B1–B3)	**±**	−	**+**	−	**+**	**+**
Subventricular zone	−	−	**+**	**+**	**±**	−

*Ascending projections*: the most conspicuous and dense terminal innervation was found in the amygdala (Fig. 3c), where a strong accumulation of axon varicosities was observed with a particularly dense distribution in the central and basolateral components of the amygdala (Table 3). A somewhat similar dense innervation was visible within the extended amygdala, such as the bed nucleus of the stria terminalis (BST) (Fig. 3d). In contrast, despite the presence of ascending fiber tracts in the medial septum, no terminal innervation was visible in the lateral septal (LS) nuclei (Fig. 3d).

A dense innervation also reached the cerebral cortex, with a particular concentration in its rostral and ventrolateral parts including the orbital cortex and olfactory-related brain areas, in particular the piriform cortex (Fig. 3c), the anterior olfactory nuclei (AON) (Fig. 4b) and the mitral and granular layers of the olfactory bulb (Fig. 4a). Innervation extended into the lateral parietal and frontal isocortex in the primary somatosensory (Fig. 3a) and motor cortices (Fig. 3b). In the medial prefrontal cortex, varicose fibers were visible mainly in the infralimbic cortex (Fig. 3a), whereas the dorsal part and the cingulate cortex were more sparingly innervated (Fig. 3b). In contrast, the caudal parts of the cortex (posterior parietal, temporal and occipital) did not seem to receive any substantial innervation of the targeted B7v sites (data not shown).

In the thalamus, innervation was essentially concentrated in the midline thalamic nuclei: the centromedian, reuniens (Re), and submedius nucleus (sub) (Fig. 3f), with hardly no innervation of the more lateral somatosensory thalamic relay nuclei (the ventrobasal or posterior thalamus) or lateral geniculate nuclei (dLGN, vLGN) (Fig. 3g). The habenula was only moderately innervated, with terminal fibers mainly in the lateral habenula (LHb) (Fig. 3f). Strikingly, and contrasting with the heavy ascending fiber tract in the mfb, the hypothalamic nuclei received hardly no innervation (Fig. 3h). Similarly the hippocampus contained only few fibers that were essentially localized in the stratum lacunosum-moleculare (Fig. 3e) of both the dorsal and ventral hippocampus. However, hardly any innervation was noted in the dentate gyrus.

In the mesencephalon, innervation was particularly conspicuous in the substantia nigra pars reticulata with moderate innervation of the ventral periaqueductal gray (PAG) and the superior colliculus (not illustrated).

A substantial descending innervation to the brainstem was noted in both cases. This terminal innervation was particularly conspicuous in lateral parts of the brainstem: the parabrachialis nucleus (PB) (Fig. 3j), with visible innervation to the trigeminal (SP5), facial (7N), and vagus (10N) nuclei (Fig. 3k). Innervation to the other monoaminergic cell groups was essentially concentrated in the locus coeruleus (LC) (Fig. 3j).

### Lateral wings of the DR (B7l) (Fig. 5) (Table 3)

**Fig. 5:**
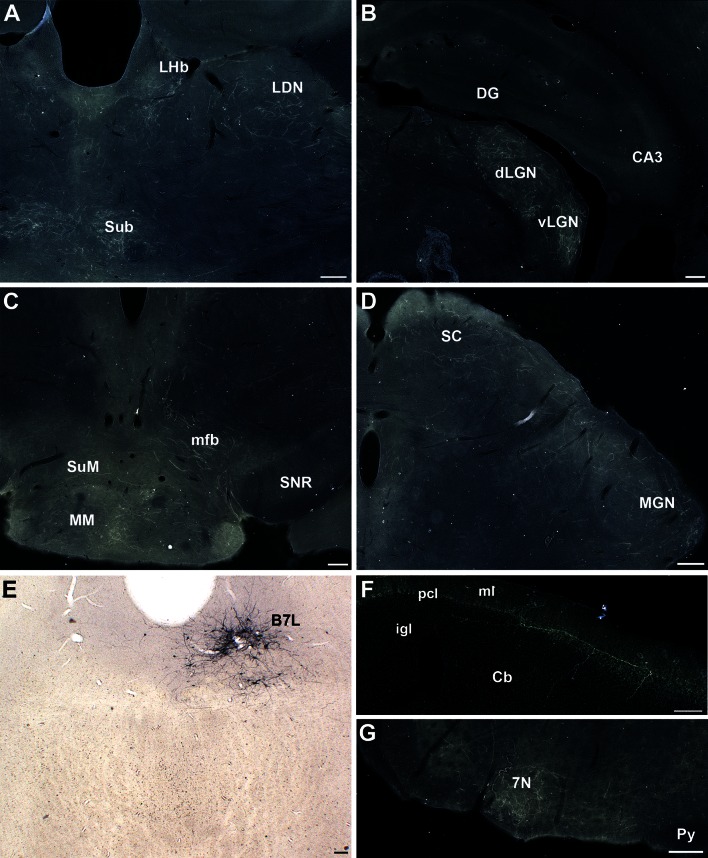
Representative 5-HT projections from the B7l- lateral wings (Case S97). GFP-transfected 5-HT neurons are shown in **e**. Main projection sites are the thalamus (**a**, **b**, **d**), the mammillary bodies (**c**), the superior colliculus (**d**) and the facial cranial nerve (7N) (**g**). A few fibers are also observed in the Purkinje (pcl) and granular cell (*igl*) layers of the cerebellum (**f**). *Scale bars* are 200 μm (**a**–**d**, **f**, **g**) and 100 μm (**e**)

The case chosen to describe the B7l projection (S97) showed an exclusive labeling of a small group of 5-HT neurons in the lateral wings of the DR (Figs. 2b, 5e). Ascending 5-HT innervation to the forebrain in this case was essentially concentrated in the thalamus, predominating in its more lateral components throughout its rostrocaudal extent (Fig. 5a, b). A sizeable projection, in consideration to the number of labeled cells was visible in the ipsilateral lateral and medial geniculate nuclei (Fig. 5b, d), and bilaterally in the parasubthalamic nucleus and the LHb (Fig. 5a). Projections were also visible in the hypothalamus mainly in the ipsilateral mammillary/supramammillary components (Fig. 5c), and in the lateral hypothalamic cell groups. In contrast, no axon terminals were found in the hippocampus (Fig. 5b) nor the septum and very sparse innervations were seen in the cerebral cortex. In the brainstem, terminal axons were essentially found in the superior (Fig. 5d) and inferior colliculi, in the cochlear, motor trigeminal, and facial nerve (Fig. 5g) nuclei. Few branched varicose fibers, indicative of terminal innervation, were also noticed in the Purkinje and granular cell layers of the cerebellum (Fig. 5f).

### Dorsal component of the DR (B7d-l) (Fig. 6) (Table 3)

**Fig. 6:**
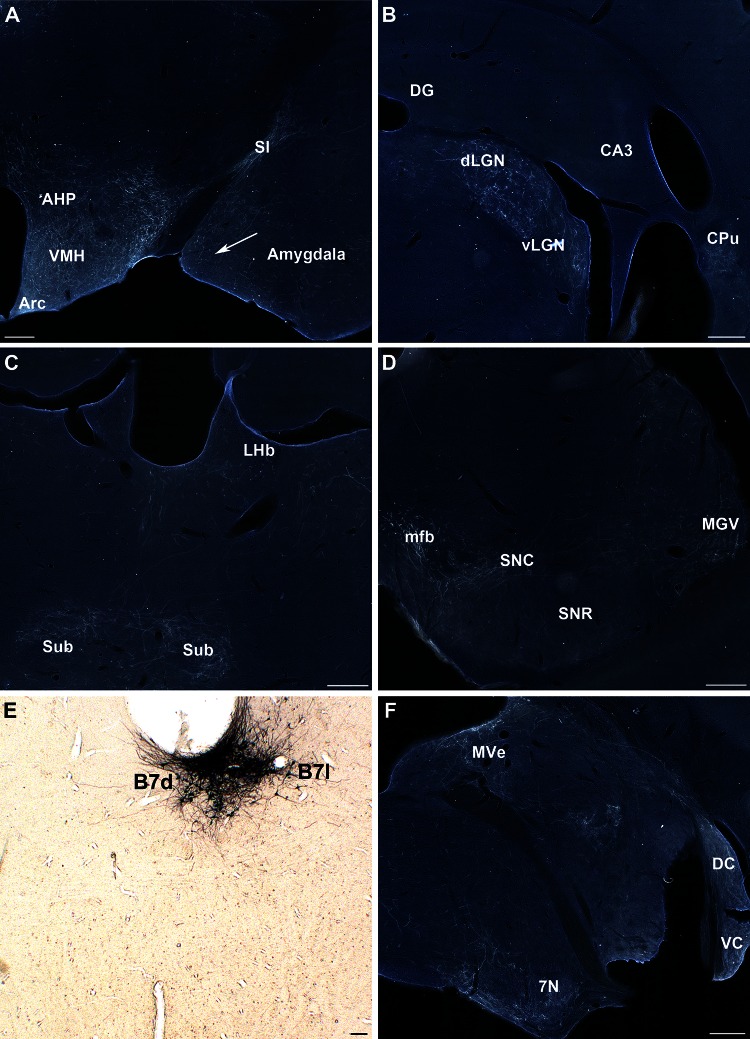
Representative 5-HT projections from the B7d-l (Case S77). GFP-transfected 5-HT neurons in the B7d-l are shown in e and main projections are observed in the hypothalamus (**a**), the thalamus (**b**, **c**), the midbrain (**d**), and hindbrain (**f**). **a** Note that B7d innervation to the amygdala is restricted to the medial amygdaloid nuclei (*arrow*). In addition, a bundle of fibers is detected in the substantia innominata (*SI*). **b** No innervation is visible in the hippocampus whereas a strong focus of innervation is visible in the caudate (*Cpu*) and the lateral geniculate nuclei (*dLGN*, *vLGN*). **c** innervation is noted in the submedius thalamic nucleus (*Sub*) and in the Lateral habenula (*LHb*). **d** In the mesencephalon innervation of the substantia nigra is limited to the pars compacta (*SNC*), and does not reach the reticulata (*SNR*). **f** In the brainstem, innervation is visible in the medial vestibular area (*Mve*), the cochlear (*DC*, *VC*) and the facial (7N) nerve nuclei. *Scale bars* are 200 μm (**a**–**d**, **f**) and 100 μm (**e**)

In 4 cases, GFP-labeled 5-HT neurons targeted the B7d and a variable portion of the B7l (S3, S77, S78, S79). We describe and illustrate the representative case S77. Ascending projections collected ventrally and bilaterally towards the mlf, spreading laterally to collect above the substantia nigra pars compacta (SNC) and ventral tegmental area (VTA), to which they provide terminal innervation. The most abundant terminal innervation was localized in the hypothalamus (Fig. 6a), followed by the lateral geniculate nuclei of the thalamus (Fig. 6b), SNC (Fig. 6d) and parts of the basal ganglia, namely the nucleus accumbens, and caudal parts of the caudate-putamen (Fig. 6b) and pallidum. These projections were mainly but not exclusively ipsilateral.

In the hypothalamus, B7d-l innervation was visible in all parts of the hypothalamus, including the paraventricular thalamus (Fig. 6a), with the exception of the suprachiasmatic and the arcuate nuclei (Fig. 6a) that received no 5-HT innervation from B7. In the thalamus, besides the dLGN, vLGN (Fig. 6b) and MGN (Fig. 6d), substantial innervation was observed in the Sub (Fig. 6c), with scattered fibers in the LHb (Fig. 6c).

In contrast in the cerebral cortex, only sparse B7d-l innervation was noted that was limited to the frontal pole, in the prelimbic and orbital prefrontal cortices. In the amygdala, a moderate B7d innervation was found essentially localized to the medial components of the amygdala with the exclusion of the basolateral and central amygdala nuclear complexes (Fig. 6a).

In the brainstem, B7d-l terminal innervation was dense in the superior colliculus and the periaqueductal gray (PAG). More ventrally it was essentially concentrated in the superior olivary complex, 7N, and dorsal and ventral cochlear nuclei (DC, VC) (Fig. 6f). In addition, a substantial fiber network was noted in the subventricular ependymal complex of the fourth ventricle (Fig. 6f), although axons did not extend into the subventricular zone (SVZ) in the forebrain.

### Caudal injections DRc (B6) (Fig. 7) (Table 3)

**Fig. 7:**
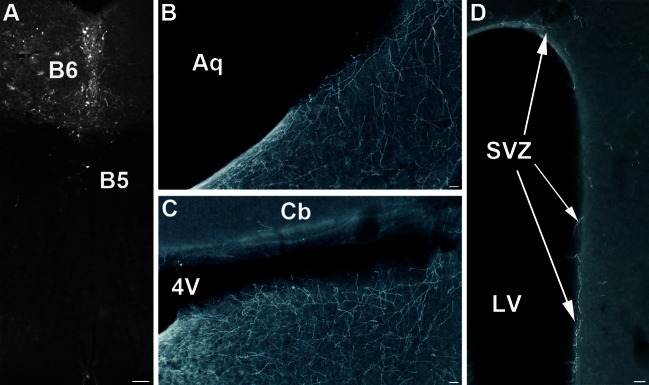
Terminal innervation of the SVZ from B6 (Case S156). **a** GFP-transfected 5-HT neurons are mainly located in the B6 nucleus. **b**, **c** innervation of the ependyma in the brainstem at the level of the 4th ventricle. **d** GFP-labeled axons are visible in the subventricular zone (*SVZ*) in the forebrain. *Scale bars* are 100 μm (**a**), 20 μm (**b**, **c**) and 50 μm (**d**). *Aq* aqueduct, *Cb* cerebellum; 4 V: fourth ventricle

The caudal part of the DR, (B6), was rarely labeled in isolation, and cases generally comprised some labeling either of the B5 (Fig. 7a) or B3 cell group. The common projections in these cases were projections to the dorsal and ventral hippocampus, the septal nuclei, and the preoptic cell groups. Projections to the amygdala were limited to the medial amygdala nuclei. Only few projections were found in the brainstem. Interestingly, however, in all the cases in which the B6 5-HT neurons were targeted, we observed substantial innervation of the subventricular 5-HT terminal network (Fig. 7b–c) as well as in the SVZ (Fig. 7d).

### MR injections (B8) (Figs. 4, 8, 9) (Table 3)

**Fig. 8:**
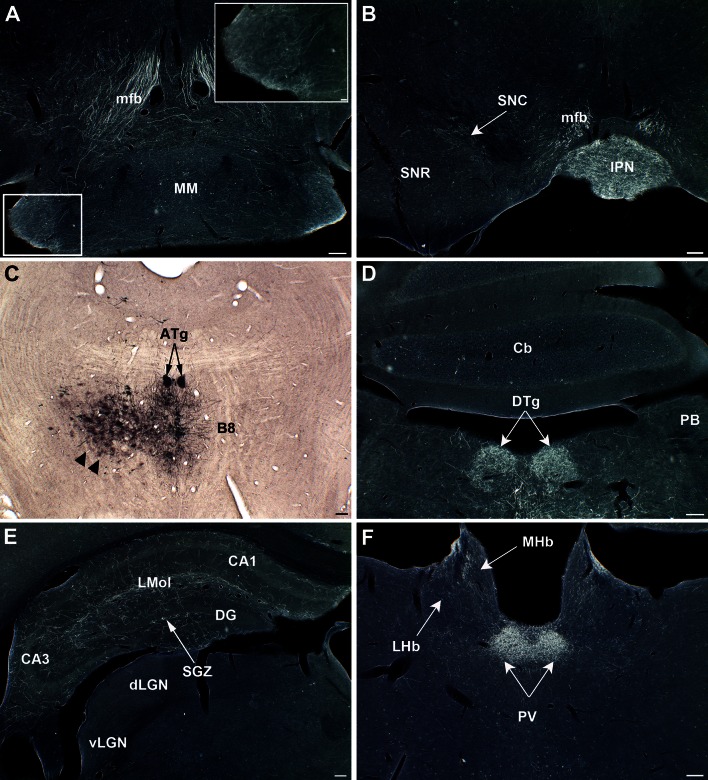
Representative 5-HT projections to the brainstem from the MR (Case S82). **a**, **b** Ascending axons from B8 course in the medial part of the mfb and provide a moderate innervation to the mammillary bodies (*MM*) with denser innervation in the lateral part (*inset*) and a strong innervation of the interpeduncular nucleus (IPN) (**b**). **c** GFP-transfected 5-HT neurons in the MR. On this picture, non-specific labeling of glial cells close to the injection site is also visible (*arrowheads*). A dense terminal innervation is noted in the anterior tegmental nuclei (*ATg*, indicated with *two arrows*). **d** Dense innervation arising from B8 targets the dorsal tegmental nucleus of Gudden (*DTg*) (*arrows*) whereas no innervation is visible in the parabrachial nucleus (*PB*). **e** GFP-terminal innervation distributes to all hippocampal terminal fields, and clear innervation of the subgranular zone (*SGZ*) of the dentate gyrus (*DG*) (*arrow*). **f** Dense terminal innervation is noted in the periventricular thalamic nuclei (*PV*, *arrows*) and in the medial habenula (*MHb*) (*arrow*). *Scale bars* 100 μm in main figures, 20 μm in the *inset* in **a**

**Fig. 9:**
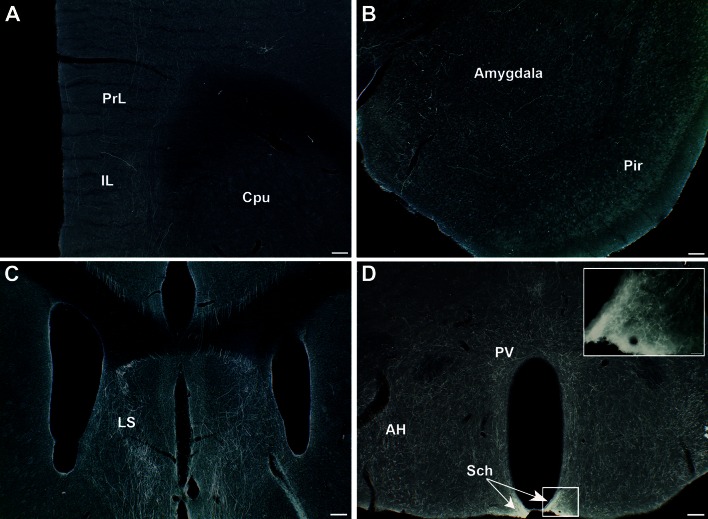
Forebrain projections from the MR. (Case S82). **a** Moderate innervation of the medial prefrontal cortex is observed in the prelimbic (*PrL*) and infralimbic (*IL*) areas, while almost none is noted in the *Cpu*. **b** Hardly no terminal innervation is observed in the amygdala or piriform cortex. **c** The lateral septal (*LS*) nuclei are densely innervated by B8 5-HT axons. **d** A dense innervation can be seen in the anterior hypothalamic (*AH*), in the magnocellular paraventricular cell groups (*PV*) and in the suprachiasmatic nucleus (*Sch*), as shown at higher magnification in the *inset*. *Scale bars* 100 μm in main figures, 20 μm in the *inset*

S82 was chosen as a representative case for description since labeled cells were limited to B8. However, additional cases with B8 + B5 labeled cells (S86, S156) showed similar patterns of innervation. The pattern of B8 projections is described from caudal (Fig. 8) to rostral (Fig. 9).

Ascending projections from B8 collected in the most medial part of the mfb and provided a bilateral terminal innervation to a number of midline brain structures from the mesencephalon to the forebrain (Fig. 8a, b). Innervation was particularly dense in the interpeduncular nucleus (Fig. 8b). An important terminal innervation was also visible in the mammillary nuclei particularly in their lateral parts (Fig. 8a), and in anterior hypothalamic areas, including the preoptic area. Very dense terminal axons were noted in the suprachiasmatic nucleus (Fig. 9d). In the dorsal thalamus, the B8 5-HT innervation was particularly concentrated in the paraventricular thalamic nuclei (PV) (Fig. 8f), whereas moderate innervation was visible in the reuniens and in midline non-specific thalamic nuclei, as well as in the medial habenula cell groups (Fig. 8f). More rostrally, B8 axons provided a very dense innervation to both the medial and lateral septal nuclei (Fig. 9c).

The B8 group provided a massive innervation to all components of the septum and hippocampus. In the latter, the innervation extended from the dorsal (Fig. 8e) to the ventral hippocampal formation. This innervation reached the hippocampal formation both from the cingulum and the fimbria-fornix pathways. Innervation was densest in the molecular and radiatum layers, extending into all hippocampal fields (Fig. 8e). A distinctive terminal-like innervation was also noted all along the subgranular cell zone (SGZ) of the dentate gyrus (DG), where adult neurogenesis is found.

In the cerebral cortex, B8 innervation was generally scattered and essentially concentrated in the dorsomedial components of the cortex: in the medial prefrontal cortex (Fig. 9a), the anterior cingulate and in the primary motor cortex. In contrast, innervation was absent or scarce in lateral parts of the cortex, such as the somatosensory or piriform cortex (Fig. 9b), as well as in the amygdala cell groups (Fig. 9b). However, in all cases with B8 labeled neurons, a conspicuous but limited patch of labeled terminals was found in the temporal cortex at the level of the rhinal fissure (data not shown).

In the olfactory bulb, a strong and selective B8 axon projection was found in the periglomerular cell layer (Fig. 4c, d). In addition, few fibers were detected in the rostral migratory stream (RMS), at the OB (Fig. 4c) and AON levels (Fig. 4d).

In the brainstem, the most remarkable concentration of 5-HT-labeled axons was found in the dorsolateral tegmental nucleus (DTg) also known as the tegmental nucleus of Gudden (Fig. 8d). This dense 5-HT innervation was visible in the mesopontine tegmental nuclei and the anterior tegmental nucleus (ATg), which are components of the DTg.

### Supralemniscal (B9) (Fig. 10)

The supralemniscal 5-HT cell group was targeted in 5 cases, three of which are illustrated in Fig. 1b. Case S83 was chosen as a prototypical case for illustration.

**Fig. 10:**
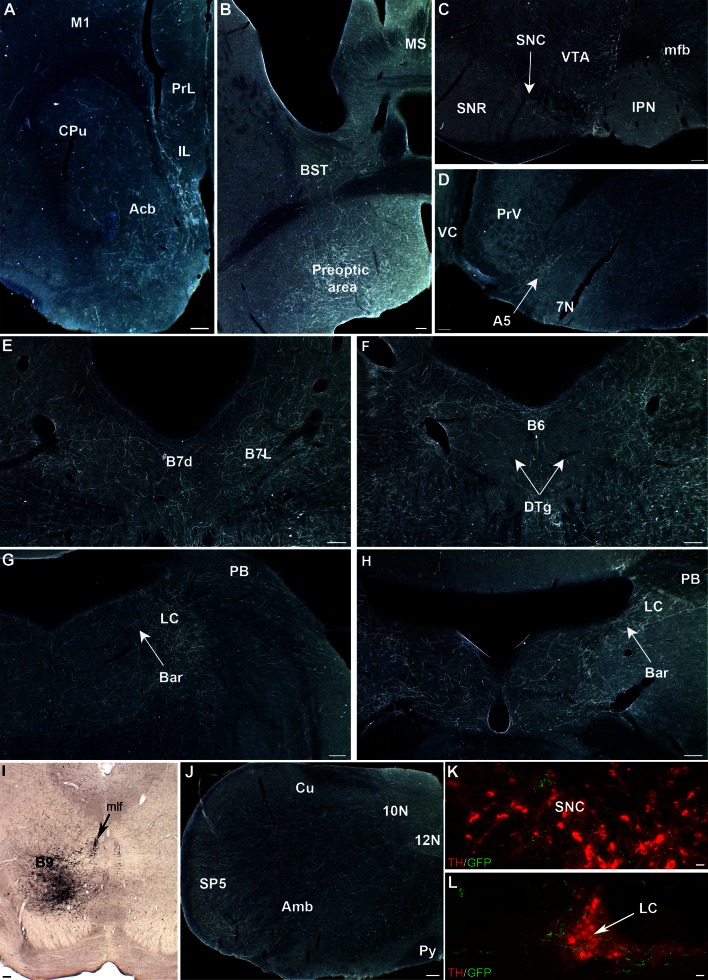
Representative 5-HT projections to the brainstem from the supralemniscal B9 nucleus. **a**, **b** Labeled axon terminals are visible in the caudate (*Cpu*), accumbens (*Acb*), bed nucleus of the stria terminalis (*BST*), and preoptic area. **c** In the midbrain, innervation is visible mainly in the VTA and substantia nigra compacta (*SNC*).** d** Labeled axon terminals are visible along the A5 noradrenergic cell group. **e**, **f** B9 innervates densely the dorsal raphe cell groups (B7 and B6), avoiding the dorsal tegmental nuclei of Gudden. **g**, **h** B9 innervation is visible in the locus coeruleus, in Barrington’s nucleus (*Bar*), as well as in the parabrachialis nucleus (PB). **i** Localization of the GFP- transfected 5-HT neurons. Axons are seen to converge towards the medial longitudinal fasciculus (*mlf*). **j** In the brainstem, a widespread network of axons is visible, covering the spinal trigeminal nucleus (*SP5*) and the nucleus Ambiguous (*Amb*). **k**, **l** Double immunostaining of GFP and tyrosine hydroxylase (*TH*) to illustrate that B9 axon terminals are in contact with dopaminergic neurons in the substantia nigra pars compacta (*SNC*) (**k**) and with noradrenergic neurons in the locus coeruleus (**l**). *Scale bars* 100 μm

Ascending projections from the B9 were relatively sparse, in comparison with the DR and MR groups: they occupied a medial and dorsal position in the mfb providing terminal innervation essentially to parts of the septum and basal ganglia. These projections were bilateral but with a predominance of innervation on the side ipsilateral to the injection site. Terminal-like innervation was observed in the medial septum (Fig. 10b), and fine terminal innervation of variable abundance according to cases was noted in the caudate-putamen, nucleus accumbens (Fig. 10a) and pallidum. Sparse innervation was also noted in the cerebral cortex and hippocampus, but with no systematic distribution. No innervation was found in the amygdala. In the hypothalamus, axon terminal innervation, when present, was mainly targeting the anterior and preoptic cell groups (Fig. 10b), and the supramammillary area. Thalamic innervation was scarce and oriented toward midline structures. In the midbrain, terminal-like fibers were visible in the SNC (Fig. 10c, k) and the VTA (Fig. 10c).

A remarkable feature of the B9 5-HT axons was the presence of descending projections towards the hindbrain. Axons collected dorsally towards the mlf in which they coursed caudally (Fig. 10i). Axons branched off from the mlf to distribute terminal fiber network in the dorsal and caudal raphe cell groups (Fig. 10e, f). A striking terminal focus was found in the LC (Fig. 10g, h, l), in Barrington’s nucleus (Fig. 10g, h), and in the area of A5 neurons (Fig. 10d). More caudally, innervation extended into the medulla providing significant input to SP5, 10N and 12N (Fig. 10j).

### Correlations between the sources of 5-HT axons (raphe subnuclei) and their targeted brain regions

**Fig. 11:**
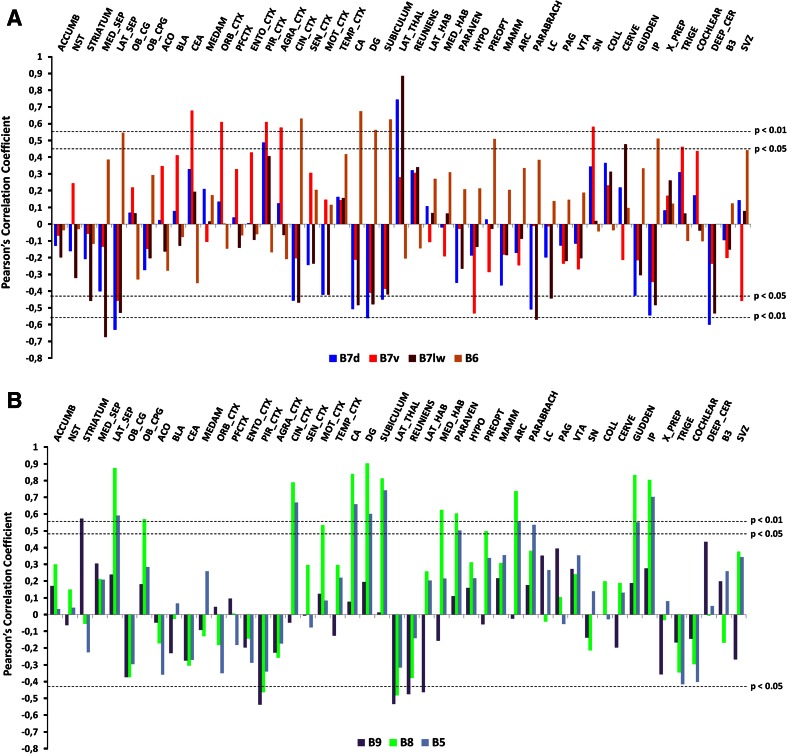
Correlation analyses between different raphe subnuclei and their target regions. The subjective rating obtained from detailed anatomical observation of each case was converted into a numerical scale (0 to 4 for the targets). The obtained semi-quantitative data from a total of 20 mice were used to calculate the Pearson’s correlation coefficient between the different sources of 5-HT axons (B5–B9) and the analyzed projecting areas. **a** Correlations between raphe subnuclei located in the dorsal pole: B7 dorsal (B7d), B7 ventral (B7v), B7 lateral wings (B7 l) and B6, with the analyzed target projecting regions. **b** Correlations between raphe subnuclei located in the ventral pole including B5, B8 and B9, with the analyzed target projecting regions. Positive and negative Pearson’s correlation coefficients reaching statistical significance are shown above or below the threshold levels (*dashed lines*), respectively. Thresholds were established at *p* < 0.05 or *p* < 0.01

To obtain a more systematic account of the topographic contribution of different 5-HT raphe subnuclei (B5–B9) in innervating specific brain regions, we performed a correlation analysis between the sources of 5-HT axon projections and their targeted brain regions. In this analysis, the subjective rating obtained from detailed anatomical observation of twenty cases (Supplementary Table 1) was converted into a numerical scale (0 to 4 for the targets) to obtain semi-quantitative data. Then, these data were used to calculate the Pearson’s correlation coefficient between different sources of 5-HT axons (B5–B9) and their projecting brain areas. (Fig. 11). This generally confirmed the description of representative cases provided above. We list below the significant correlations obtained by this analysis.

Positive correlations of the ventral part of B7 (B7v) were found with the orbital cortex (0.65, *p* < 0.001), piriform cortex (0.62, *p* < 0.01), agranular cortex (0.54, *p* < 0.05) and with the central amygdaloid nucleus (0.7, *p* < 0.001). In the mesencephalon, a positive correlation was found with the substantia nigra (0.54, *p* < 0.05). Among the descending pathways only the vestibulo-cochlear nerve showed a positive correlation (0.45, *p* < 0.05). B7v projections rarely invade the SVZ and hippocampus as indicated by their negative correlations (−0.53, *p* < 0.05 and −0.46, *p* < 0.05, respectively).

The lateral wings of B7 (B7l) showed a strong positive correlation with lateral thalamic regions (0.87, *p* < 0.001). Negative correlations were noted with medial and lateral aspects of the septum (−0.63 and −0.53, *p* < 0.01 and *p* < 0.05, respectively), and with the hippocampus (DG −0.53, *p* < 0.05). More caudally, negative correlations were also found in relationship to the interpeduncular nucleus (−0.55, *p* < 0.05) and the parabrachial nucleus (−0.49, *p* < 0.05).

The dorsal part of B7 (B7d) showed a strong positive correlation with the lateral thalamus (0.68, *p* < 0.001), with the piriform cortex (0.59, *p* < 0.01), cingulate cortex (−0.51, *p* < 0.05) and hippocampal formation (CA −0.55, *p* < 0.05, DG −0.59, *p* < 0.01, subiculum −0.49, *p* < 0.05). B7d showed negative correlations with the lateral septum (−0.67, *p* < 0.01), interpeduncular nucleus (−0.58, *p* < 0.01), nucleus of Gudden (−0.45, *p* < 0.05), mammillary nucleus (−0.45, *p* < 0.05) as well as deep structures of cerebellum (−0.56, *p* < 0.05).

B8 subnuclei projections were significantly correlated with the hippocampus: CA fields (0.83, *p* < 0.001), DG (0.76, *p* < 0.001) and subiculum (0.67, *p* < 0.001). Positive correlations were also found with the lateral septum (0.69, *p* < 0.001), the cingulate cortex (0.77, *p* < 0.001), motor cortex (0.74, *p* < 0.001), the paraventricular nucleus (0.60, *p* < 0.01), the interpeduncular nucleus (0.67, *p* < 0.01), the nucleus of Gudden (0.70, *p* < 0.01), and the medial aspects of the habenula (0.50, *p* < 0.05).

B6 projections correlated positively with the lateral septum (0.47, *p* < 0.05) and the hippocampal formation (CA 0.59, *p* < 0.01, DG 0.48, *p* < 0.05, subiculum 0.50, *p* < 0.05). Some hypothalamic regions were also correlated with B6 such as the preoptic area (0.54, *p* < 0.05). Interestingly, this analysis also revealed that B6 represents the major source of 5-HT for the SVZ (0.48, *p* < 0.05).

Additionally, the correlation analysis allowed us to discriminate projections arising from B5 that were often co-labeled together with other raphe subnuclei in our injections. This showed that B5 projection targets were very similar to those of B8 group. Positive correlations were found with the lateral septum (0.78, *p* < 0.001), the cingulate cortex (0.74, *p* < 0.001) and the hippocampal formation (CA 0.80, *p* < 0.001, DG 0.82, *p* < 0.001, subiculum 0.87, *p* < 0.001). Other positive correlations were found with the thalamic paraventricular nucleus (0.69, *p* < 0.01), preoptic area (0.45, *p* < 0.05), arcuate nucleus (0.76, *p* < 0.001), mammillary nucleus (0.45, *p* < 0.05) and the interpeduncular nucleus (0.88, *p* < 0.001). In the brainstem, the parabrachial nucleus (0.56, *p* < 0.05) and nucleus of Gudden (0.82, *p* < 0.001) showed positive correlations. Conversely, the trigeminal nucleus (−0.49, *p* < 0.05) and the vestibulo-cochlear nerve (−0.47, *p* < 0.05) showed negative correlations indicating that these areas are not targeted by B5 5-HT axons.

B9 projections showed a positive correlation with the caudate-putamen (0.57, *p* < 0.01), but was negatively correlated with the piriform cortex (−0.54, *p* < 0.05), lateral thalamus (−0.53, *p* < 0.05) and reuniens (−0.48, *p* < 0.05). Additionally, B9 projections were hardly present in the lateral habenula, showing a negative correlation (−0.46, *p* < 0.05).

## Discussion

**Fig. 12:**
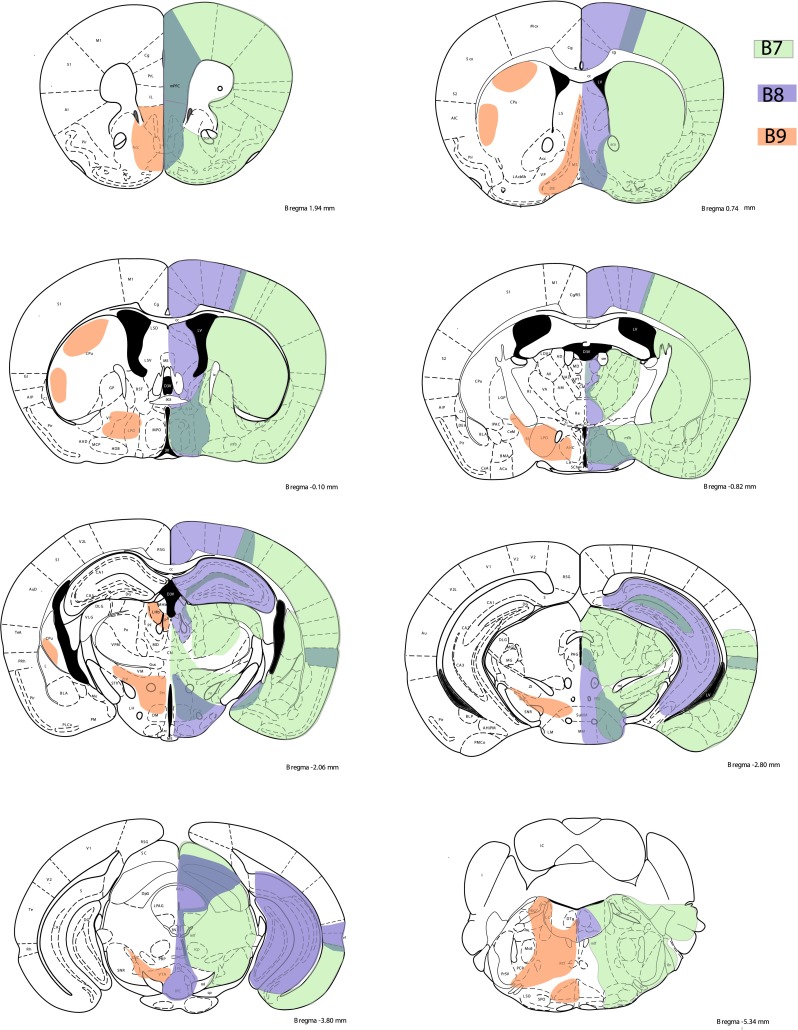
Summary scheme of coronal brain diagrams showing the main projection sites of the B7, B8 and B9 cell groups as revealed in the present analysis

**Fig. 13:**
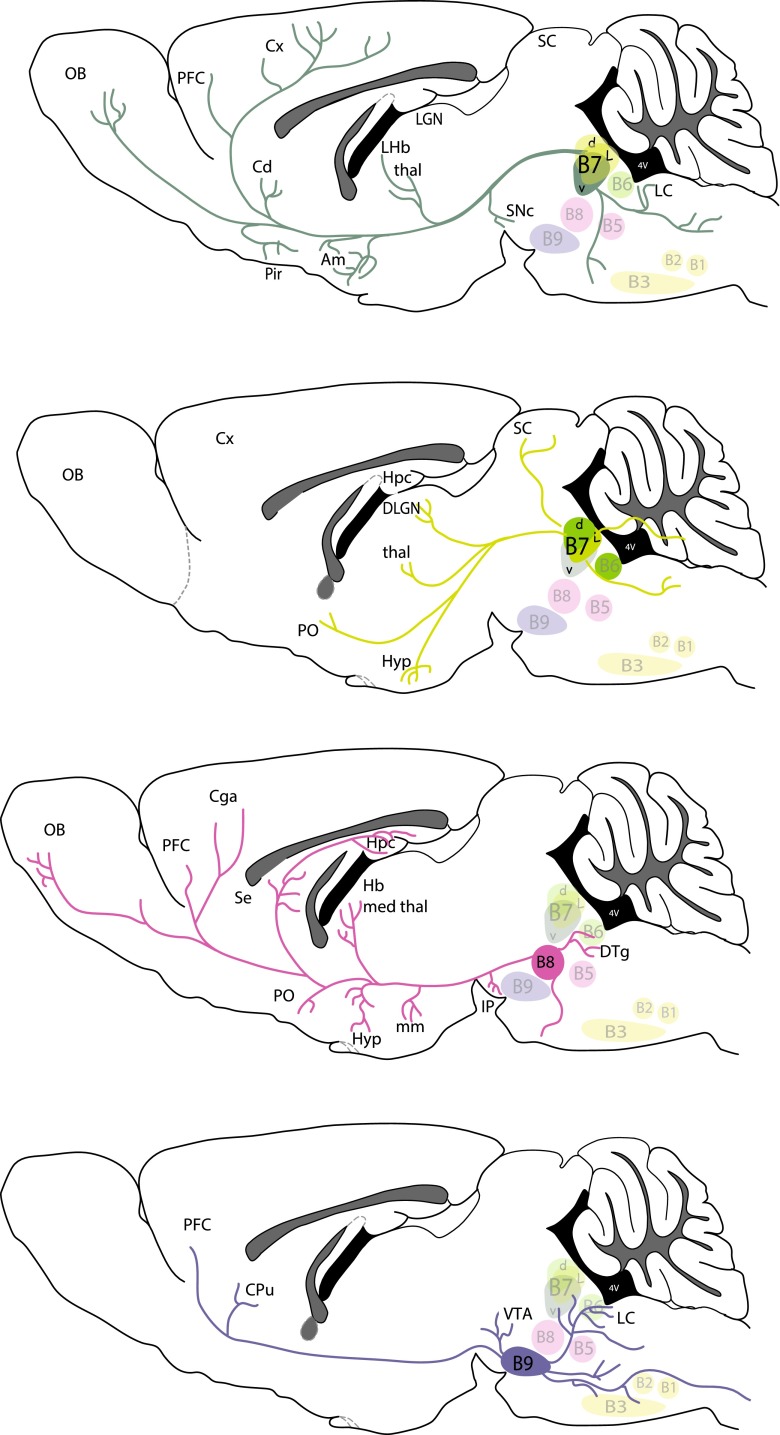
Summary scheme of the principal targets of the main B5–B9 subgroups in sagittal brain diagrams

Present results show that despite the diffuse nature of the 5-HT raphe projections, the individual raphe subnuclei have distinctive and complementary terminal fields in both the forebrain and the brainstem. These results complement and extend previous neuroanatomical studies in the field providing a comprehensive and comparative view of the entire projection of the 5-HT rostral raphe cell groups of the mouse brain. Compared to previous tracing methodologies, the present study enabled a selective anterograde labeling of the 5-HT raphe neurons. Moreover, because targeting of the tracer was limited to restricted subcomponents of the rostral 5-HT cell groups, it was possible to identify the contribution of different raphe subnuclei, to the total DR and MR projection fields (Figs. 12, 13). Finally, this approach allowed us analyzing specifically for the first time the projections that arise from more dispersed B9 5-HT neurons located in the supralemniscal areas. These technical improvements should make the present description a useful anatomical guide helping to interpret functional studies in the field of 5-HT research that increasingly make use of genetically modified mice.

The first clear organizing principle of the ascending raphe projections revealed by the present tracing experiments is the dichotomy of 5-HT projections arising from the dorsal (DR) and the median raphe (MR) cell groups. 5-HT neurons arising from the DR and MR occupy largely complementary axon terminal fields in the forebrain and hindbrain (Figs. 12, 13). For instance, the hippocampus, septum, caudal hypothalamus, mammillary bodies, interpeduncular nucleus, and mesopontine tegmental nuclei received their major inputs from the MR neurons, while the amygdala, cerebral cortex, striatum, substantia nigra, locus coeruleus, and trigeminal nerve nuclei receive their major inputs from the DR neurons. Although several brain regions showed an overlap in the 5-HT inputs from these two sources, more detailed analyses of the distribution of the DR and MR terminal fields showed some degree of laminar specificity as exemplified for instance in the olfactory bulb and the habenular nuclei (see discussion below). Generally, the MR ascending pathways and the brain targets innervated by MR 5-HT neurons were located within midline cerebral structures, while the DR axons showed more lateral localizations (Fig. 12). Such a medio-lateral distribution of the MR *vs.* DR projections has already been noted in early studies performed in rats and cats. In their seminal description, Jacobs et al. (Jacobs et al. 1974) established a distinction between the DR and MR forebrain targets using a lesion approach in rats; this showed that 5-HT reductions in the hippocampus, striatum and cortex differed after DR or MR lesions (Jacobs et al. 1974). Anterograde tracing with tritiated leucine in cats (Bobillier et al. 1975, 1979) and rats (Azmitia and Segal 1978) showed a topographical distinction of the ascending tracts issued from the DR and MR, with the MR tracts being localized more medially in the mfb than the DR pathways. More precise anterograde anatomical tracing, obtained with phaseolus (Vertes 1991; Vertes et al. 1999), allowed to describe the main terminal domains of the DR and the MR, and indicated the complementarity of these innervations. Interestingly, although these earlier anterograde tracing studies labeled indistinctly the 5-HT and non-5-HT axon terminals, the distribution pattern of the 5-HT containing terminal depicted in the present account shows a very similar pattern, with a few exceptions in the habenula and mesopontine nuclei that are detailed below. This is coherent with studies using retrograde tracing experiments in combination with 5-HT immunocytochemistry, where raphe neuron projections to a given brain area were most generally found to comprise a mix of 5-HT and non-5-HT neurons (Jackson et al. 2009; Kim et al. 2004; Kiyasova et al. 2011; Kohler and Steinbusch 1982; Steinbusch et al. 1981). Some of these non-5-HT neurons of the DR and MR are long distance projecting axons, and recent studies have shown that their neurochemical identity could be glutamatergic (Hioki et al. 2009; Jackson et al. 2009) or GABAergic (Bang and Commons 2012). Overall, these results tend to indicate that there are topographic rules guiding the distribution of the DR and MR projection fields, which are largely independent of the neurochemical identity of the raphe neurons.

A noteworthy novel observation revealed in the present study is the existence of a robust and specific MR 5-HT innervation to different components of the mesopontine tegmental nuclei. Both the dorsal and ventral tegmental nuclei of Gudden (DTg and VTg, respectively) contain a remarkably dense 5-HT terminal innervation of beaded varicosities that was identified here as being entirely provided by the MR raphe groups. This MR projection went unnoticed in previous anterograde tracing studies (Azmitia and Segal 1978; Bobillier et al. 1979; Vertes et al. 1999), but was illustrated in more recent genetic tracing studies (Bang et al. 2011), where DTg innervation was noted as originating from raphe cell groups of the R3–R5 rhombomeres. Interestingly, the GABAergic neurons that constitute these nuclei have been shown to express high levels of the 5-HT1A receptors (Bonnavion et al. 2010) indicating they are likely under a strong inhibitory 5-HT drive from the MR. Interest in DTg and VTg circuits stems from anatomical observations showing that they are specifically and reciprocally interconnected with the mammillary bodies, and involved in the “Papez” memory circuit (Vann 2013). Additionally, these pontine tegmental nuclei have been implicated in the modulation of hippocampal theta rhythms (Kocsis et al. 2001) and shown to be required for spatial learning, both processes that are also controlled by 5-HT (Kocsis et al. 2006; Richter-Levin and Segal 1991; Staubli and Xu 1995). Lesions of the VTg produce memory deficits similar to those observed after hippocampal or mammillary lesions (Vann 2009). The fact that these interconnected structures receive a common and strong MR input further points to include them in a functional ensemble involved in memory processing.

The complementarity of the DR and MR innervations in a given brain structure is of particular interest to consider, highlighting the different regulatory processes that could be driven by the different raphe cell groups. The habenular nuclei are an example of this, given their implication in behavioral responses to pain, anxiety, stress and sleep (Hikosaka 2010), all processes that are under 5-HT modulatory control. The present study revealed that 5-HT innervation to the lateral habenula (LHb) is provided by the DR cell groups, whereas 5-HT innervation to the medial habenula (MHb) is provided by the MR. Interestingly, the DR receives regulatory feedback from the LHb, whereas the MR receives inputs from the MHb; this is indicative of the existence of different and yet closely juxtaposed functional ensembles linking habenular and raphe subnuclei. Of note, the LHb provides a major inhibitory control on the DR neurons (Sego et al. 2014) that could underlie depression-like symptoms noticed in neurological diseases (Sourani et al. 2012). In contrast, the MHb, which is proposed to mediate the behavioral effects of nicotine (Salas et al. 2009), exerts a strong cholinergic control on the interpeduncular nucleus, which in turn provides an inhibitory feedback on the MR (Hsu et al. 2013). It will thus be of interest to further dissect out the functional implications of these different raphe-habenular pathways in reward-related behaviors, using more selective tools that individually target specific subcomponents of these neural circuits.

Complementarity of the DR and MR innervation was also particularly striking in the olfactory bulb (OB), suggesting a possible implication in different functional regulatory loops. While MR innervation was conspicuously localized in the glomerular cell layer, offering a perfect anatomical substrate for a direct control of primary olfactory inputs (Petzold et al. 2009), the DR innervation was restricted to the granule cell layer in a position to be likely implicated in the control of secondary olfactory processing. The importance of DR 5-HT in the secondary olfactory control is further supported by the dense DR innervation of secondary olfactory relays including the anterior olfactory nuclei and the piriform cortex (Datiche et al. 1995). Another interesting aspect of 5-HT innervation to the OB is the existence of a rhythmic release of 5-HT in the OB (Corthell et al. 2013). The OB is one of the few identified structures showing an internal circadian oscillator, which control for olfactory responses independently of the suprachiasmatic nucleus (Abraham et al. 2005). Our results showing that B8 neurons are the 5-HT source of both the glomerular cell layer and the suprachiasmatic nucleus suggest that MR 5-HT could play a role in the modulation of circadian clocks in both structures.

Other brain structures that receive terminal innervation from both the DR and MR are the pallial-derived structures such as the cerebral cortex and hippocampus. Our tracing analyses showed that the majority of the cortical 5-HT innervation, in particular to the piriform, orbitofrontal, and primary somatosensory cortices, arises from the DR, with only scarce contribution from the MR. While there is a moderate cortical innervation from the MR, it is essentially restricted to medial cortical regions such as the anterior cingulate and motor cortices. One exception is the presence of dense but limited patches of 5-HT terminals arising from the MR in the perirhinal cortex. Conversely, the dorsal and ventral aspects of the hippocampal formation were found to receive all their main 5-HT inputs from the MR, with only scarce innervation from the DR. This topographic distribution is in agreement with previous observations in rats using phaseolus anterograde tracing (Vertes 1991; Vertes et al. 1999). It, however, differs from previous descriptions that were based on the analysis of the fine morphology of 5-HT-labeled axon terminals (Kosofsky and Molliver 1987) and their differential vulnerability to neurotoxics (Mamounas et al. 1991). In the latter studies, two classes of 5-HT axon terminals were identified in the cerebral cortex and hippocampus: D fibers with small fusiform and M fibers with large spherical varicosities originating from the DR and MR, respectively. This led to a proposed distribution of cortical DR/MR innervation that only partly matches the present observations. In the present study, anterogradely labeled terminals showed the typical D and M-type morphologies. However, they were not unequivocally linked to a single raphe subnucleus, and appeared to be more related to the brain region examined. For instance, in the hippocampus and septum, both D and M varicosities were found to derive from MR neurons. Notably, the subventricular and ventricular 5-HT plexus contains large 5-HT varicosities classically associated with the MR (Mamounas et al. 1991), however, we found that this innervation originated almost exclusively from the caudal DR (B6). These latter findings are consistent with illustration shown in a recent study analyzing the role of 5-HT inputs on stem cells in the SVZ (Tong et al. 2014). The fact that axon morphology is related to brain region targeted more than to the raphe subnucleus of origin coincides also with recent observations of Gagnon and Parent (2014) analyzing the morphology of single reconstructed neurons from the DR.

However, more detailed analysis are warranted to settle this issue since there was a good agreement between fiber morphology and their subnuclear origin, in the case of the OB and perirhinal cortex, where the distribution of M-type axon terminals matched the distribution of MR afferents (Mamounas et al. 1991).

Besides the MR/DR organization, a further topographic organization of the 5-HT neuron projections was found along the dorsoventral, rostrocaudal and medio-lateral axes. These different anatomical projections are likely to contribute to differences in the functional properties of distinct raphe subnuclei after the exposure of animals to different stressors (Commons 2008; Hale and Lowry 2011; Spannuth et al. 2011). Most strikingly the dorsal and ventral part of the DR differed in their projections to the hypothalamus: the B7d innervates several hypothalamic and preoptic nuclei that were avoided by the B7v raphe axons. Reciprocally, the B7v massively innervated neocortical structures that were almost entirely devoid of B7d inputs. Along the lateral axis, the main characteristic of the 5-HT projections from B7l, was a selective input to the lateral and medial geniculate nuclei, suggesting a specific role for B7l, also known as the “lateral wings” in sensory processing, as also suggested in previous studies (Vasudeva et al. 2011). Interestingly, raphe neurons located in the B7l have been shown to have a higher excitability than the other raphe neurons (Crawford et al. 2010). However, the most conspicuous differences in projection patterns within the DR were found along the rostrocaudal dimension: the B6 showed projections resembling those originating from the MR. For instance 5-HT neurons from B6 send projections to the hippocampus and the lateral septal nuclei, whereas B7 neurons rarely target this structure. Conversely, B7 but not B6 neurons send projections to the cerebral cortex and caudate-putamen. These observations are consistent with previous conventional tracing studies (Imai et al. 1986) showing that projections from the rostral raphe subnuclei reach the caudate-putamen, while projections to the hippocampus arise from the caudal raphe subnuclei. Moreover, functional studies show that caudal/rostral DR cell groups are differentially activated in pathophysiology. For instance, the B6 is selectively activated after nicotine withdrawal (Sperling and Commons 2011), and shows increases of TPH2 mRNA expression in postmortem studies of suicide subjects (Bach-Mizrachi et al. 2008). Interestingly the B7 and B6 cell groups appear to have different afferent inputs (KG Commons personal communication). Overall, our tracing analysis confirms that B7 is not a uniform cell group but consists of different subcomponents with distinctive anatomical profiles, as clarified in the present analysis. Thus, although a given B7 axon can send collaterals to the cortex, caudate-putamen and hippocampus (Gagnon and Parent 2014), at a population level, the highly collateralized neurons may represent a small subset of neurons, while most DR raphe neurons show a topographic organization of the brain areas targeted by individual components.

Contrasting with the wealth of studies on the DR and MR groups, the B9 5-HT cell group has been much less studied, as it is fairly loosely distributed away from the midline. However, studies in primates (Baker et al. 1991) and rodents (Vertes and Crane 1997) revealed the numerical importance of this 5-HT cell group which comprises about 20–25 % of the total mesopontine 5-HT neuronal subpopulation. Although tracing studies have reported the presence of retrogradely labeled neurons in B9 after tracer injections in the cerebral cortex (O’Hearn and Molliver 1984) or septum (Jones and Cuello 1989), there are no systematic studies analyzing the projection pattern of this group. The present study selectively analyzed the supralemniscal cell groups, showing that besides the ascending projections to the caudate-putamen, cortex and hippocampus, the majority of the projections were directed caudally into the brainstem, with noteworthy inputs to the other 5-HT cell group within the DR and MR, and noradrenergic neurons in the locus coeruleus. The supralemniscal nuclei could, therefore, be a major player in intrinsic raphe connectivity.

Interestingly, genetic studies in mice showed altered distributions of 5-HT-labeled axons in the forebrain and brainstem, further emphasizing the heterogeneity of neurons in terms of their projection patterns (Kiyasova and Gaspar 2011). In mutations affecting axon outgrowth or guidance, 5-HT axons appeared to essentially fail to reach cortical structures (Donovan et al. 2002), or did not invade their terminal target zones (Katori et al. 2009). However, in mutations of the transcription factor Pet1 which acts to specify 5-HT raphe neurons (Hendricks et al. 2003), a small fraction of the 5-HT raphe neurons were maintained in the DR and MR with highly specific brain targets (Kiyasova et al. 2011), that correlate in the present study essentially with the targets of the B7v, and with a subset of the MR projections.

## Conclusions

The present mapping of projections obtained after sparse labeling of different cell groups in serotonin rostral raphe subnuclei showed that there are topographical rules for the projections along the three major axes of the brainstem, rostrocaudal, medio-lateral and dorsoventral. Thus, although a given raphe subgroup sends broad axon projections to the forebrain and brainstem, there is a defined topographic organization of these projections, with some very striking specific targets in particular arising from the MR cell groups. The topography of projections relates to the cytoarchitectonic delimitations previously established in the raphe, and follows in part the rhombomeric organization of the raphe that has been recently described. However, the developmental origin from different rhombomeric territories does not suffice to explain all the characteristics of the topographic organization that is observed within the DR (B7–B6) cell groups. Therefore, it is likely that, similar to other brain circuits, a combination of axon guidance molecules both attractive and repulsive is involved, to guide 5-HT raphe neuron axons to their proper brain targets.

## Electronic supplementary material

Below is the link to the electronic supplementary material.

Supplementary Table 1: Semi-quantitative estimate of the distribution of the GFP- transfected 5-HT neurons in the different raphe subnuclei in the 20 cases used for the Pearson’s correlation analysis. The number of GFP-labeled neurons was scored on serial sections through the raphe identifying in each case, the number of cells in the different raphe subnuclei (B5-B9). The score was as following: 0 = 0-5 neurons; 1 = 5-20 neurons; 2 = 20-50 neurons; 3 = > 50 neurons. These scores were then used for correlation analyses. Scores obtained in each case were correlated to the semi-quantitative rating of density of terminal innervation in selected brain regions. The Pearson correlation coefficient between the different sources of 5-HT axons and their projecting areas is shown in Fig. 11 for the different raphe subnuclei analyzed: B5, B6, B7 dorsal (B7d), B7 ventral (B7v), B7 lateral wings (B7l), B8 and B9. Access to the original data, of these cases is available on the following link “http://1drv.ms/1y2FNst”. The files of the scanned slides are as a “.ndpi” format and can be imaged with the NDP -viewer (free download) (DOCX 20 kb)

Supplementary material 2 (TIFF 1.63 GB)

Supplementary material 3 (NDPI 643 MB)

Supplementary material 4 (ZIP 1.68 GB)

Supplementary material 5 (ZIP 103 MB)

Supplementary material 6 (ZIP 1.25 GB)

Supplementary material 7 (ZIP 1.76 GB)

## Abbreviations

5-HT: 5-Hydroxytryptamine, serotonin
AADC: Aromatic l-amino acid decarboxylase
AAV: Adeno-associated virus
Acb: Accumbens
AHA: Anterior hypothalamic area
Am: Amygdala
AON: Anterior olfactory nucleus
Arc: Arcuate nucleus
Bar: Barrington nucleus
BST: Bed nucleus of the stria terminalis
Cga: Anterior cingulate cortex
CPu: Caudate-putamen nucleus
Cx: Cortex
DC: Dorsal cochlear nucleus
DG: Dentate gyrus
dLG: Dorsal lateral geniculate nucleus
DR: Dorsal raphe nucleus = B7 + B6
DRc: Caudal DR = B6
DRd: Dorsal DR = B7d
DRL: Lateral DR = B7l
DTg: Dorsal tegmental nucleus of Gudden
VTg: Ventral tegmental nucleus of Gudden
GFP: Green fluorescent protein
GCL: Granular cell layer of the olfactory bulb
GL: Glomerular layer of the olfactory bulb
EPL: External plexiform layer of the olfactory bulb
Hip: Hippocampus
Hy: Hypothalamus
IL: Infralimbic cortex
LDN: Laterodorsal thalamic nucleus
LMol: Lacunosum moleculare (hippocampus)
PAG: Periaqueductal gray
SERT: Serotonin transporter
IL: Infralimbic cortex
IPN: Interpeduncular nucleus
LC: Locus coeruleus
LDTg: Laterodorsal tegmental nucleus
LHb: Lateral habenula
LS: Lateral septum
M1, M2: Motor cortex
MGN: Medial geniculate nucleus
MHb: Medial habenula
mfb: Medial forebrain bundle
mlf: Medial longitudinal fasciculus
Mve: Medial vestibular nucleus
NTS: Nucleus of tractus solitarius
OB: Olfactory bulb
PaM: Paraventricular hypothalamic nucleus
PB: Parabrachialis nucleus
PFC: Prefrontal cortex
Pr: Nucleus prepositus
PrV: Trigeminal nucleus, principalis
PrL: Prelimbic cortex
Pir: Piriform cortex
PO: Preoptic area
PV: Paraventricular thalamic nucleus
RMTg: Rostromedial tegmental nucleus
RSA: Retrosplenial area
RMS: Rostral migratory stream
Sch: Suprachiasmatic nucleus
Se: Septum
SNC: Substantia nigra, compact part
SNR: Substantia nigra, reticular part
Sp5: Spinal trigeminal nucleus
Sub: Submedius thalamic nucleus
SVZ: Subventricular zone
vLGN: Ventral geniculate nucleus
VC: Ventral cochlear nucleus
VTA: Ventral tegmental area
