# Supplementary material for: Conditional anterograde tracing reveals distinct targeting of individual serotonin cell groups (B5–B9) to the forebrain and brainstem
Source: Brain Struct Funct. 2014 Nov 18;221(1):535–61. doi: 10.1007/s00429-014-0924-4 (PMC4750555; doi:10.1007/s00429-014-0924-4)
Supplement: Supplementary file 1 — Supplementary Table 1: Semi-quantitative estimate of the distribution of the GFP- transfected 5-HT neurons in the different raphe subnuclei in the 20 cases used for the Pearson’s correlation analysis. The number of GFP-labeled neurons was scored on serial sections through the raphe identifying in each case, the number of cells in the different raphe subnuclei (B5-B9). The score was as following: 0 = 0-5 neurons; 1 = 5-20 neurons; 2 = 20-50 neurons; 3 = > 50 neurons. These scores were then used for correlation analyses. Scores obtained in each case were correlated to the semi-quantitative rating of density of terminal innervation in selected brain regions. The Pearson correlation coefficient between the different sources of 5-HT axons and their projecting areas is shown in Fig. 11 for the different raphe subnuclei analyzed: B5, B6, B7 dorsal (B7d), B7 ventral (B7v), B7 lateral wings (B7l), B8 and B9. Access to the original data, of these cases is available on the following link “http://1drv.ms/1y2FNst”. The files of the scanned slides are as a “.ndpi” format and can be imaged with the NDP -viewer (free download) (DOCX 20 kb) [file 429_2014_924_MOESM1_ESM.docx]

|  | ***Raphe Subnuclei*** | | | | | | |
| --- | --- | --- | --- | --- | --- | --- | --- |
| ***Mouse*** | **B7d** | **B7v** | **B7lw** | **B9** | **B8** | **B6** | **B5** |
| **S3** | **3** | **2** | **3** | **0** | **0** | **0** | **0** |
| **S4** | **3** | **3** | **3** | **0** | **0** | **0** | **0** |
| **S52** | **1** | **3** | **0** | **0** | **0** | **0** | **0** |
| **S53** | **0** | **3** | **0** | **0** | **0** | **0** | **0** |
| **S63** | **3** | **3** | **3** | **0** | **0** | **2** | **0** |
| **S64** | **3** | **3** | **3** | **0** | **0** | **1** | **0** |
| **S77** | **3** | **0** | **3** | **0** | **0** | **1** | **0** |
| **S78** | **2** | **0** | **3** | **0** | **0** | **1** | **0** |
| **S79** | **3** | **0** | **3** | **0** | **0** | **1** | **0** |
| **S82** | **0** | **1** | **1** | **3** | **3** | **1** | **0** |
| **S83** | **0** | **0** | **0** | **3** | **1** | **0** | **0** |
| **S84** | **0** | **1** | **0** | **3** | **0** | **0** | **0** |
| **S86** | **0** | **1** | **1** | **1** | **3** | **1** | **2** |
| **S97** | **0** | **0** | **3** | **0** | **0** | **0** | **0** |
| **S101** | **0** | **0** | **0** | **0** | **0** | **3** | **3** |
| **S110** | **0** | **1** | **0** | **3** | **0** | **0** | **0** |
| **S111** | **1** | **0** | **1** | **3** | **0** | **0** | **0** |
| **S146** | **0** | **0** | **0** | **0** | **3** | **3** | **0** |
| **S156** | **0** | **0** | **0** | **0** | **3** | **3** | **3** |
| **S163** | **0** | **0** | **0** | **0** | **3** | **3** | **3** |
